# Neuroscience, Ethics, and National Security: The State of the Art

**DOI:** 10.1371/journal.pbio.1001289

**Published:** 2012-03-20

**Authors:** Michael N. Tennison, Jonathan D. Moreno

**Affiliations:** 1 Wake Forest University, Winston-Salem, North Carolina, United States of America; 2 University of Pennsylvania, Philadelphia, Pennsylvania, United States of America

## Abstract

National security organizations in the United States, including the armed services and the intelligence community, have developed a close relationship with the scientific establishment. The latest technology often fuels warfighting and counter-intelligence capacities, providing the tactical advantages thought necessary to maintain geopolitical dominance and national security. Neuroscience has emerged as a prominent focus within this milieu, annually receiving hundreds of millions of Department of Defense dollars. Its role in national security operations raises ethical issues that need to be addressed to ensure the pragmatic synthesis of ethical accountability and national security.

## Abstract 2

Military involvement and research in neuroscience generates unique ethical, legal, and social issues that require careful elucidation and consideration in order to align the potentially conflicting needs of national defense, public interest, and scientific progress.

## Introduction

During the past decade, the US national security establishment has come to see neuroscience as a promising and integral component of its 21st century needs. Much neuroscience is “dual use” research, asking questions and developing technologies that are of both military and civilian interest. Historically, dual use has often involved a trickle down of military technology into civilian hands. The Internet, for example, originated as a non-local, distributed means to secure military information. In the case of neuroscience, however, civilian research has outpaced that of the military. Both National Research Council (NRC) reports and Department of Defense (DoD) funding reveal ongoing national security interests in neuroscience and indicate that the military is quite eager to glean what it can from the emerging science [1],[2]. To pursue cognitive neuroscience research, the Pentagon's science agency, the Defense Advanced Research Projects Agency (DARPA), received about US$240 million for the fiscal year of 2011, while the Army trails at US$55 million, the Navy at US$34 million, and the Air Force at US$24 million [3].

The military establishment's interest in understanding, developing, and exploiting neuroscience generates a tension in its relationship with science: the goals of national security and the goals of science may conflict. The latter employs rigorous standards of validation in the expansion of knowledge, while the former depends on the most promising deployable solutions for the defense of the nation. As a result, the exciting potential of high-tech developments on the horizon may be overhyped, misunderstood, or worse: they could be deployed before sufficiently validated.

Current state-of-the-art neuroscience, including new forms of brain scanning, brain–computer interfaces (BCIs), and neuromodulation, is being tapped for warfighter enhancement, deception detection, and other cutting-edge military applications to serve national security interests.

## Brain–Computer Interfaces

BCIs exemplify the dual use nature of neuroscience applications. BCIs convert neural activity into input for technological mechanisms, from communication devices to prosthetics. The military's interests in BCIs are manifold, including treatment modalities, augmented systems for controlling vehicles, and assistance for detecting danger on the battlefield.

In the late 1990s, scientists demonstrated neurological control of the movement of a simple device in rats, and soon thereafter, of a robotic arm in monkeys [4]. More recently, a pilot study of BrainGate technology, an intracortical microelectrode array implanted in human subjects, confirmed 1,000 days of continuous, successful neurological control of a mouse cursor [5]. Non-invasive technologies for harnessing brain activity also show promise for human use. Progress has recently been reported on a “dry” EEG cap that does not require a gel to obtain sufficient data from the brain. The “brain cap” is reported to reconstruct movements of humans' ankle, knee, and hip joints during treadmill walking in order to aid rehabilitation [6].

DARPA's Augmented Cognition (AugCog) program sought to find ways to use neurological information gathered from warfighters to modify their equipment accordingly. For example, the “cognitive cockpit” concept involved recording a pilot's brain activity to customize the cockpit to that individual's needs in real time, from selecting the least burdened sensory organ for communicating information to prioritizing informational needs and eliminating distractions [7]. Although the Augmented Cognition moniker (and funding mechanism) seem to have been dropped, its spirit lives on in other DARPA projects. For example, the Cognitive Technology Threat Warning System is developing portable binoculars that convert subconscious, neurological responses to danger into consciously available information [8]. Such a system could reduce the information-processing burden on warfighters, helping them to identify and respond to areas of interest in the visual field more quickly.

Via intracortical microstimulation (ICMS), a neurologically controlled prosthetic could send tactile information back to the brain in nearly real time, essentially creating a “brain-machine-brain interface” [9]. The technology underlying this concept is already evolving, and some researchers hope that optogenetics, which both enables “precise, millisecond control of specific neurons” and “eliminates most of the key problems with ICMS,” will ultimately supplant the ICMS for sensory feedback [9]. In addition to devising prosthetics that can supply sensory information to the brain, brain-machine-brain interfaces may directly modify neurological activity. Portable technologies like near infrared spectroscopy (NIRS), for example, could detect deficiencies in a warfighter's neurological processes and feed that information into a device utilizing in-helmet or in-vehicle transcranial magnetic stimulation (TMS) to suppress or enhance individual brain functions [2].

Much of the technological evolution of warfare has introduced a distance between the parties involved. From the advent of firearms to airplanes, aerial bombs to remotely operated drones, the visceral reality of combat afforded by the physical proximity to one's enemy has steadily eroded. In 2007, researchers taught a monkey to neurologically control a walking robot on the other side of the world by means of electrochemical measurements of motor cortical activity [9]. Considering this in light of the work on robotic tactile feedback, it is easy to imagine a new phase of warfare in which ground troops become obsolete.

## Warfighter Enhancement

The therapeutic paradigm of medical practice aims to heal and reduce suffering, to return the ill to a state of normal health. Yet, many interventions can be used by the healthy to enhance specific traits or capacities beyond the physiological or statistical norm [10]. For example, BCIs can operate prosthetics for therapeutic purposes, but they could also connect to orthotic exoskeletons that enhance strength and endurance. Similarly, therapeutic drugs like methylphenidate can help patients recover focus and attention, but they are also used, for example, by healthy college students looking to maximize academic performance [11]. Whether they do in fact improve performance is open to disagreement [11],[12]. Military pharmaceutical neuroenhancement came to the public's attention in 2003 when “two American pilots accidentally killed four Canadian soldiers and injured eight others in Afghanistan” [13]. It turned out that the pilots had been taking Dexedrine, the amphetamine-based “go pills” often used to reduce the fatigue induced by long missions.

In 2008, a report for the US Army compared the effects of amphetamines with those of modafinil, a drug typically used and approved to treat narcolepsy, in combination with sleep-aiding drugs. Despite the controversy over “go pills”, the study found that for long-duration missions, both amphetamines and modafinil have statistically similar effects of reducing the cognitive decline associated with fatigue [14]. Other reports state that modafinil significantly outperforms methylphenidate for cognitive enhancement in healthy individuals, “especially on people undergoing sleep deprivation” [15]. Related research has investigated other ways to combat fatigue as well. Published in 2007, a DARPA-sponsored study showed that nasally administered orexin-A, a neuropeptide, restored the short-term memory of sleep-deprived monkeys [16].

In its 2009 report for the US Army, the NRC recommends that TMS should also be a part of further research on central nervous system fatigue [2]. Studies suggest that TMS can enhance a variety of neurological functions in healthy individuals, from mood and social cognition to working memory and learning [17]. Another noninvasive neuromodulation technology, transcranial pulsed ultrasound, was demonstrated to have a number of promising effects, from being “useful for sonoporation in gene therapy” to “promoting nerve regeneration” [18]. With the aid of both DARPA and US Army funding, researchers envision and work toward developing portable, in-helmet ultrasound transducers capable of stimulating neural circuits with a better precision and depth than TMS [19]. Direct current polarization, or transcranial direct current stimulation (TDCS), is another noninvasive, DARPA-supported technology for neuromodulation. “As might be expected, TDCS can enhance cognitive processes occurring in targeted brain areas” [20], including learning and memory [17].

While cognitive augmentation will enhance performance on some tasks, other situations call for the reduction of neurological capacity. For example, if a memory of a traumatic event could be dampened, one may be less likely to experience post-traumatic stress disorder (PTSD) as a result. In 2002, scientists produced preliminary evidence that propranolol, when administered shortly after a traumatic event, could mitigate the long-term potential for internal cues to invoke post-traumatic stress [21]. More recently, scientists demonstrated that propranolol can similarly reduce PTSD symptoms when administered “after retrieval of the memory of a past traumatic event”, not just immediately after the event itself [22].

Human enhancement may benefit individuals and society in myriad ways, but it also poses many risks. In the civilian world, if more and more people begin enhancing their minds and bodies, individuals may eventually feel subtly coerced into enhancing themselves in order to remain competitive in school or the workplace [10]. In the military context, the risk of coercion is much more pronounced [13]:

According to the Uniform Code of Military Justice, soldiers are required to accept medical interventions that make them fit for duty. Experimental treatments are a harder case, but the US government has shown a tendency to defer to commanders in a combat situation if they think some treatment is likely to do more harm than good, even if unproven.

If a warfighter is allowed no autonomous freedom to accept or decline an enhancement intervention, and the intervention in question is as invasive as remote brain control, then the ethical implications are immense. As Peter W. Singer has observed, “the Pentagon's real-world record with things like the aboveground testing of atomic bombs, Agent Orange, and Gulf War syndrome certainly doesn't inspire the greatest confidence among the first generation of soldiers involved [in human enhancement]” [23].

## Neuroscientific Deception Detection and Interrogation

National security agencies are also mining neuroscience for ways to advance interrogation methods and the detection of deception. The increasing sophistication of brain-reading neurotechnologies has led many to investigate their potential applications for lie detection. Deception has long been associated with empirically measurable correlates, arguably originating nearly a century ago with research into blood pressure [24]. Yet blood pressure, among other modern bases for polygraphy like heart and breathing rates, indicates the presence of a proxy for deception: stress. Although the polygraph performs better than chance, it does not reliably and accurately indicate the presence of deception, and it is susceptible to counter measures. Because of these problems with the polygraph, researchers are eagerly following up on preliminary successes in using new neurotechnological modalities for detecting deception.

“Brain fingerprinting” utilizes EEG to detect the P300 wave, an event-related potential (ERP) associated with the perception of a recognized, meaningful stimulus, and it is thought to hold potential for confirming the presence of “concealed information” [25]. The technology is marketed for a number of uses: “national security, medical diagnostics, advertising, insurance fraud and in the criminal justice system” [26]. Similarly, fMRI-based lie detection services are currently offered by several companies, including No Lie MRI [27] and Cephos [28]. DARPA funded the pioneering research that showed how deception involves a more complex array of neurological processes than truth-telling, and that fMRI arguably can detect the difference between the two [29]. No Lie MRI also has ties to national security: they market their services to the DoD, Department of Homeland Security, and the intelligence community, among other potential customers [30].

The Defense Intelligency Agency (DIA)-commissioned 2008 NRC report, *Emerging Cognitive Neuroscience and Related Technologies*, in which one of the present authors (JDM) participated, reiterates the conclusion of a 2003 NRC report [31] that “traditional measures of deception detection technology have proven to be insufficiently accurate” [1]. While the NRC ultimately recommends pursuing “research on multimodal methodological approaches for detecting and measuring neurophysiological indicators of psychological states and intentions”, it cautions that like traditional polygraphy, neurological measurements do not directly reveal psychological states [1]. In fact, many scholars and scientists dispute the validity of brain scan-based lie detection [24],[32].

In addition to questions of scientific validity, these technologies raise legal and ethical issues. Legally required brain scans arguably violate “the guarantee against self-incrimination” because they differ from acceptable forms of bodily evidence, such as fingerprints or blood samples, in an important way: they are not simply physical, hard evidence, but evidence that is intimately linked to the defendant's mind [32]. Under US law, brain-scanning technologies might also raise implications for the Fourth Amendment, calling into question whether they constitute an unreasonable search and seizure [33].

Another neuroscientific field stimulating national security interest pertains to the hormone oxytocin, which has been shown to augment the expression of various virtues, from “trust and trustworthiness” to “generosity and sacrifice” [34]. Without elaborating, the NRC's 2008 report specifies oxytocin as a “neuropeptide of interest” [1]. If the interest in question relates to pharmacologically incapacitating the psychological defenses of interrogation suspects, this may conflict with the Chemical Weapons Convention (CWC). According to the CWC, a chemical that can cause “temporary incapacitation” is defined as a “toxic chemical” and is therefore banned from such use [35]. Beyond this ethical concern, oxytocin is far from being confirmed as a truth serum, and without further verification it should not be treated as such. The history of research on finding the ultimate truth serum is long and storied. Suffice it to say, “[T]he urban myth of the drugged detainee imparting pristine nuggets of intelligence is firmly rooted and hard to dispel” [36].

## Recommendations

This paper has detailed the national security establishment's interest in and ability to fund a panoply of diverse neuroscientific studies. It has also reviewed the ethical, legal, and social issues that emerge from this relationship. Yet, discussions in themselves will not ensure that the translation of basic science into deployed product will proceed ethically or contribute to the greater good. These considerations must be embedded and explored at various levels in society: upstream in the minds and goals of scientists, downstream in the creation of advisory bodies, and broadly in the public at large.

Although they may receive funding from national security agencies, neuroscientists may not consider how their work contributes to warfare. As we have seen, however, neuroscience does, and will continue to, play a role in military operations. This fact spawns a plenitude of ethical concerns, from which one may surmise that the sciences should divorce themselves from the military completely. However, the fact that the material explored in this paper is public information speaks to the possibility that a discussion about the role and limits of neuroscience in national security may be open and transparent. Bifurcating public science from national security may only drive the same research underground, undermining its current public accountability [13]. Thus, it would be impractical to try to circumvent the ethical problems simply by cutting ties between science and national defense.

Many would agree with George Mason University anthropologist Hugh Gusterson that “[m]ost rational human beings would believe that if we could have a world where nobody does military neuroscience, we'll all be better off. But for some people in the Pentagon, it's too delicious to ignore” [37]. In any case, as we have suggested, the dual use possibilities for neuroscience render such a world unlikely. Therefore, scientists themselves could become more aware of the dual use phenomenon, whether their work is specifically funded by national security bodies or not, in order to create a more self-conscious scientific enterprise. They could also involve themselves in constructing the parameters to guide and govern their relationships with national security agencies. Just as many nuclear scientists opposed the development of atomic weapons, contributing to the test-ban treaties of the 1960s and the drawdown of armed missiles in the 1980s [13], neuroscientists could consider and promulgate their perspectives on the military implications and ethical issues associated with their work.

## Abbreviations

BCI: brain–computer interface
DARPA: Defense Advanced Research Projects Agency
NRC: National Research Council
TMS: transcranial magnetic stimulation
